# Effects of school-based interventions on motivation towards physical activity in children and adolescents: protocol for a systematic review

**DOI:** 10.1186/s13643-019-1029-1

**Published:** 2019-05-10

**Authors:** Yolanda Demetriou, Anne K. Reimers, Marianna Alesi, Lidia Scifo, Carla Chicau Borrego, Diogo Monteiro, Anne Kelso

**Affiliations:** 1 Department of Sport and Health Sciences, Technical University of Munich, Georg-Brauchle-Ring 62, 80992 Munich, Germany; 2 Department of Sport Science and Sport, Friedrich Alexander University Erlangen-Nuremberg, Erlangen, Germany; 3 Department of Psychology, Educational Science and Human Movement, University of Palermo, Palermo, Italy; 4 Sport Sciences School of Rio Maior, Polytechnic Institute of Santarém Research Center in Life Quality(CIEQV), Santarém, Portugal; 5 Sport Sciences School of Rio Maior, Polytechnic Institute of Santarém Research Center in Sport, Health and Human Development (CIDESD), Vila Real, Portugal

**Keywords:** PRISMA-P, Girls, Boys, Randomized controlled trial, Youth, Cochrane, Exercise, Students

## Abstract

**Background:**

Recent studies have observed low levels of physical activity in children and adolescents worldwide. Physical activity interventions are increasingly carried out to counteract this development. The school environment is an ideal setting for such interventions to take place as large numbers of children and adolescents can be addressed. With the assumption that motivation is the key to initiate and sustain beneficial health behaviors, theory-based intervention studies apply motivational strategies to increase students’ participation in physical activity. The main objective of this systematic review will be to analyze the effects of school-based physical activity interventions on a variety of motivational outcomes towards physical activity in school-aged children and adolescents.

**Methods:**

Comprehensive literature searches will be conducted in multiple electronic databases, including MEDLINE, Scopus, PsycINFO, ERIC, PSYNDEX, Physical Education Index, and SPORTDiscus. We will include randomized controlled trials (RCTs) and quasi-experimental studies examining the effects of school-based physical activity interventions (e.g., physical activity components during school lessons including physical education, or during morning, lunch and afternoon breaks). Primarily extracurricular physical activity interventions will not be considered. The primary outcomes will be students’ motivation, basic psychological needs, goal orientation, enjoyment, and motivational teaching climate in physical education. Secondary outcomes will be the students’ physical activity behaviors in-class, during school, and in leisure time. Only peer-reviewed articles published in English will be considered. Three reviewers will independently screen all citations and full-text articles, and two reviewers will abstract data. The quality of the included studies will be assessed with the Cochrane Collaboration’s tool for assessing risk of bias for RCTs and the GRADE methodology will be used to assess the certainty of the body of retreived evidence.

**Discussion:**

In order to increase and maintain physical activity levels in children and adolescents, motivation towards physical activity should be sustained. It is anticipated that the results of this systematic review will provide information as to which strategies implemented in the school setting are effective in increasing students’ motivation towards physical activity, and hence increase their physical activity during school and after-school hours.

**Systematic review registration:**

PROSPERO CRD42018110306

**Electronic supplementary material:**

The online version of this article (10.1186/s13643-019-1029-1) contains supplementary material, which is available to authorized users.

## Background

Participating in regular exercise and physical activity (PA) has been shown to benefit physical, mental, and social health as well as academic performance [1–4]. The World Health Organization recommends 60 min of moderate-to-vigorous physical activity (MVPA) per day for children and adolescents aged 5–17 years [5]. However, based on self-report data, in most European countries, less than 50% of children and adolescents follow these guidelines [6]. An analysis of device-based measured global PA data of 13–15-year-old adolescents, collected from 105 countries, revealed that 80.3% did not meet the recommended activity guidelines [7].

A school setting is ideal for implementing interventions aimed to increase PA levels because entire groups of children and adolescents, regardless of their socio-economic or migration status, can be addressed [8]. Although physical education (PE) offers children and adolescents an opportunity to be physically active [1], decreased levels of PA in PE have been observed, accompanied by lower physical fitness levels in children and adolescents [9]. Strategies to promote PA during PE have shown to increase overall time spent being physically active as well as participation in PE [10]. School-based PA interventions were found to not only positively affect PA and motor performance, but also benefit health outcomes and exercise knowledge in children and adolescents [10–12]. In a previous study, members of our research team found the school-based intervention effects on PA behavior to be partially mediated by self-efficacy [11]. A detailed evaluation of mediating factors of energy balance-related behavior found evidence for a mediated effect of self-regulation, enjoyment, intrinsic motivation, and autonomy support on physical activity [13]. Since mediating variables explain the causal sequence between the intervention and the outcome, the identification of relevant mediators can increase effectiveness of future interventions [13]. Intervention developers should therefore disclose the theoretical base of their intervention including the mediating variables along with the applied intervention strategies. Only then is it possible to draw conclusions as to which strategies are effective in changing mediators of a certain behavior [13].

Several theoretical frameworks exist on which interventions to promote physical activity can be based on. The self-determination theory (SDT) [14] has been found to be an appropriate theoretical framework for developing health intervention programs and for understanding the motivations of children and adolescents towards PA [15, 16]. When implementing the SDT in health intervention studies, the focus is directed “on the processes through which a person acquires the motivation for initiating new health-related behaviours and maintaining them over time” [17]. Within SDT, different types of motivation can be characterized along the autonomy-control continuum, depending upon the degree to which autonomous or controlled regulations are represented [18]. Autonomous behavior is characterized by the extent to which an individual agrees, and is willing, to engage in a certain behavior, i.e., the degree to which an individual is intrinsically motivated to act. Intrinsically motivated behavior is accompanied by a feeling of effectance and enjoyment [18]. Controlled behavior, on the other hand, is led by a consequence of external reward, social approval, or avoidance of punishment. Yet, extrinsically motivated behavior can also be experienced as autonomous when the behavior is associated with valued or important outcomes [18]. Hence, behavior can be externally regulated (controlled by external forces the individual does not agree with) or can be controlled through introjection, in which case an individual acts to receive self- or external approval, and avoid feelings of guilt, shame and disapproval [17, 18]. Amotivation can be described as a state of passiveness, a lack of intentionality and motivation to act, and can be based on a feeling of lack of competence or arise from a lack of interest, relevance, or value in a given action [14, 18].

With respect to a targeted behavior, SDT assumes that the development of intrinsic motivation requires the satisfaction of three basic psychological needs: autonomy, competence, and relatedness [14, 17]. Regarding health behavior, autonomy and competence are crucial for the processes of internalization and integration of health-beneficial behaviors like PA [14, 17]. Creating an autonomy supportive environment can increase adherence and health outcomes [14, 17]. Furthermore, for individuals to adopt health-beneficial behaviors and its values, a sense of belonging needs to be met [17]. Satisfaction of these psychological needs has been associated with better health-related outcomes, such as increased PA [17].

Building upon the SDT, the Hierarchical Model of Intrinsic and Extrinsic Motivation by Vallerand [19] considers intrinsic motivation, extrinsic motivation, and amotivation on a situational, contextual, and global level. According to this theoretical model, motivation varies both in its type and in its level of generality. Specifically, situational motivation can be described as motivation during a specific task in PE, while contextual motivation can be described as motivation towards PE in general. Global motivation describes the general motivation personality. The different types of motivation exist within the individual at the three different levels of generality [19]. Almolda et al. [20] assume that interventions successful in changing situational motivation could affect contextual motivation (i.e., physical education in general) and finally global motivation, leading to a healthy lifestyle.

Another theory, focusing on classroom motivation, is the Achievement Goal Theory, where goal orientations are grouped into performance goal orientation and mastery goal orientation. Performance goal orientation is characterized by being successful without much effort, outperforming others and being judged based upon ability. Mastery goal orientation is characterized by developing new skills, valuing the process of learning and attainment of mastery [21]. Ames and Archer [21] reported that students perceiving an emphasis on mastery goals used effective learning strategies, preferred challenging tasks, and had a more positive attitude towards class. Perception of performance goal orientations were negatively related to attitudes and self-perception of ability [21]. In the context of PE, Goudas and Biddle [22] found that the mastery dimension was present not only when self-improvement was valued and effort was rewarded rather than ability, but also when students felt the teacher took personal interest in them and were involved in decision-making.

Motivational teaching strategies to increase students’ motivation in PE have been developed and include amongst others the Task, Authority, Recognition, Grouping, Evaluation, Time (TARGET) approach [23, 24]; the Sport Education Model [25]; and Teaching Games for Understanding (TGfU) approach [26].

Systematic reviews have pointed out that the effects of PA interventions within a school setting on PA behavior are for the most part small, for example, an increase in 2.6 min of PE-related PA [10, 11, 27]. Additionally, the effects on PA behavior may decline in the long term, due to a decrease in motivational constructs [28]. Inconclusive results were found when examining the effects of school-based PA interventions on enjoyment, which is linked to intrinsic motivation [18]. Dudley et al. [27] was not able to determine effects of PA interventions on enjoyment outcomes due to poor methodological quality of the reviewed studies and their statistical power. The findings of a meta-analysis by Burns et al. [28] indicated significant effects of school-based intervention strategies on PA enjoyment, with a true population effect of small-to-moderate magnitude. Their results were also limited due to the methodological heterogeneity of the examined studies [28]. A meta-analysis conducted by Braithwaite, Spray [29], showed that motivational climate interventions in PE had small overall treatment effects (*g* = 0.103) in groups exposed to mastery motivational climates [29]. The largest treatment effects were found for behavioral outcomes (*g* = 0.39–0.49), followed by affective (e.g., attitudes, dedication, enjoyment) (*g* = − 0.27 to 0.59), and cognitive outcomes (e.g., anxiety, competence, perception of motivational climate, ability and effort) (*g* = − 0.25 to 0.32) [29].

In summary, the introduced reviews and meta-analyses suggest that theory-based PA interventions [13, 15, 16] implementing motivational strategies may have positive treatment effects on PA behavior and outcomes associated with motivation [11, 27–29]. However, none of these studies evaluated PA intervention effects on the different types of motivation, which are assumed to determine the direction of PA behavior [18, 30]. Therefore, the main objective of this systematic review is to analyze the effects of school-based PA interventions on a variety of motivational outcomes towards PA in school-aged children and adolescents.

## Methods

This systematic review protocol was registered in the PROSPERO international prospective register of systematic reviews (registration number CRD42018110306) and prepared in accordance with the Preferred Reporting Items for Systematic Reviews and Meta-Analysis Protocols (PRISMA-P) 2015 statement [31] (Additional file 1). The final review will be reported using the Preferred Reporting Items for Systematic Reviews and Meta-Analysis (PRISMA) statement as guidance [32]. Relevant changes of the protocol will be documented and published within the results of the final review.

### Eligibility criteria

This systematic review will include any randomized controlled trial (parallel group or cluster-randomized) as well as quasi-experimental trial examining the effects of school-based PA interventions on motivation towards PA. The PA components must be offered either in addition to regular PE or as modified PE classes (e.g., using motivational teaching approaches in PE) implemented primarily during the school day, i.e., regular school hours (PE), morning, lunch, or afternoon breaks. Intervention components being delivered primarily after school or outside the school setting and community-based programs will not be considered. PA components can be provided by PE or classroom teachers, external staff (e.g., members of the research group), or through web-based applications, internet, and video games. Eligible studies should focus on children and adolescents aged 6–9 years without any known health issues. Studies primarily targeting populations with specific health conditions, such as overweight or obesity, will be excluded. Comparators will be either a control group not receiving any additional PA components or an active control group participating in regular PE classes. Only studies published in the English language in peer-reviewed journals will be included.

### Outcomes of interest

Primary outcomes of interest include: students’ intrinsic and extrinsic, amotivation, basic psychological needs (autonomy, competence, relatedness), goal orientations, enjoyment, and the motivational climate in PE (task or performance climate). The studies had to report at least one of these outcomes quantified using questionnaires, e.g., the Physical Activity Enjoyment Scale (PACES) [33], Learning and Performance Orientations in PE Classes Questionnaire (LAPOPECQ) [34], or Basic Psychological Needs in Exercise Scale (BPNES) [35].

Secondary outcomes of interest are physical activity levels, step counts, exercise frequency, and exercise duration measured by using questionnaires, accelerometers, pedometers, or direct observation in the three domains: in-class, during the school day, and in leisure time. Examples of questionnaires examining students’ PA behavior are the 7-day physical activity recall (PAR) questionnaire [36] or the Leisure-Time Exercise Questionnaire (LTEQ) [37].

In order to enable an analysis of intervention effects based on change scores from baseline, outcome data should be collected at baseline and post-test (or follow-up) using the measurement tools described above. Ideally, mean values and standard deviations of the outcomes should be reported.

### Search strategy

The primary source of literature will be a structured search of seven electronic databases: Scopus (Elsevier), ERIC (EBSCO), MEDLINE (Ovid), PsycINFO (EBSCO), PSYNDEX (EBSCO), Physical Education Index (ProQuest), and SportDiscus (EBSCO). The search will include a broad range of terms and keywords related to children/adolescents, school-based interventions, physical activity, and motivation (Table 1). A draft search strategy for MEDLINE (Ovid) is provided in Additional file 2.

**Table 1:** PICO categories and keywords used for study identification

Category	Keywords
Population	Children; youth; adolescents; students; pupils; boys; girls
Intervention	Intervention; training; experiment; program; education; treatment; evaluation
	School; physical education; lesson
	Physical activity; exercise; sport; movement; cycling; walking
Outcome	Motivation; intrinsic motivation; extrinsic motivation; amotivation; basic psychological needs; motivational climate; enjoyment
Study design	Experimental/quasi-experimental design

### Study selection

All articles identified from the literature search will be screened by at least two reviewers independently (YD, AK, and/or DR). First, titles and abstracts of articles returned from initial searches will be screened based on the eligibility criteria outlined above. Second, full texts will be examined in detail and screened for eligibility. Third, references of all considered articles will be hand-searched to identify any relevant report missed in the search strategy. Any disagreements will be resolved by discussion to meet a consensus, if necessary. Records will be managed with EndNote x8 (Clarivate Analytics, Philadelphia, PA, USA).

### Data extraction

Two reviewers (AK, DR) will systematically extract study details independently using a piloted data extraction form without the reviewers being blinded to the authors and journals. Details on publication information, study design, study population (i.e., participants’ characteristics), intervention methods (intervention content for the control and intervention group including the duration, frequency, intensity, and content of the PA components delivered to the students), theoretical background, measurement instruments, and intervention outcomes (i.e., results on effectiveness of intervention on motivation towards PA and PA outcomes) will be entered into the spreadsheet. If information is missing or clarification of data is required, authors will be contacted via e-mail. A maximum of two contact attempts will be made.

### Quality assessment

For the assessment of the risk of bias of the primary studies, we will use the Cochrane Collaboration’s tool for assessing risk of bias in randomized trials [38, 39]. Two reviewers will independently evaluate methodological quality (AK, DR). Any uncertainties or disagreements will be discussed with a third reviewer (YD). The tool is a domain-based evaluation, in which critical assessments will be made separately for sequence generation (selection bias), allocation sequence concealment (selection bias), blinding of participants and personnel (performance bias), blinding of outcome assessment (detection bias), incomplete outcome data (attrition bias), selective outcome reporting (reporting bias), and other potential sources of bias. The judgment for each entry will involve assessing the risk of bias as “low risk,” as “high risk,” or as “unclear risk,” with the last category indicating either lack of information or uncertainty about the potential for bias. Judgements of risk of bias will be represented graphically within and across studies in each domain.

The certainty of evidence across studies will be assessed for each outcome as high, moderate, low, or very low using the GRADE approach [40]. With the GRADE approach, RCT evidence starts at the highest certainty level but may be downgraded based on an assessment of the following domains: study limitations (risk of bias), imprecision, heterogeneity, indirectness, and suspicion of publication bias. GRADEpro|GDT software (©2015, McMaster University and Evidence Prime, Inc.) will be used to create a summary of findings table and rate the certainty of the evidence using the GRADE framework. Additionally, we will discuss and draw conclusions on how the elements assessed by GRADE may affect the confidence we have in the study’s results.

### Data synthesis

It is anticipated that the included studies in this systematic review will have a diverse range of research methods (e.g., regarding study design, intervention characteristics, setting, measurement methods, participants’ characteristics, outcome measures). Therefore, using meta-analysis to integrate and summarize the included studies is unlikely to be appropriate. Instead, a narrative synthesis of results will be conducted. We will descriptively summarize effect estimates using the reported effect sizes, confidence intervals, and *p* values for primary and secondary outcomes. Summary tables describing the studies, their results, and methodological quality will be provided, and additional information will be provided in the text. The included studies and their findings are planned to be grouped according to students’ age and motivational outcomes measured. The results will be evaluated under consideration of the theoretical framework, intervention strategies, and methodological quality to determine the effectiveness of PA interventions on students’ motivation towards PA.

Additionally, intervention results on PA behavior will be grouped according to the domains in which PA was assessed, e.g., in PE classes or in leisure time. Similar to the analysis of motivational outcomes, the findings will be analyzed considering the theoretical framework, intervention strategies, students’ age, and methodological quality. In this respect, the aim is to determine which intervention strategies are also effective in changing PA behavior.

## Discussion

This protocol presents justification for, and planned methods of, a systematic review to examine the effectiveness of school-based PA interventions on a variety of motivational outcomes towards PA. We will consider the strengths and limitations of the identified evidence as well as those of our review, and we will discuss the findings in the context of other relevant reviews. Potential limitations of this review could include the restriction to a narrative data synthesis, which could result in an overestimation of intervention effects, as well as the restriction to English language. In case changes to the protocol will be made, these will be described in the full systematic review.

Nonetheless, this systematic review can provide information as to which strategies are effective in increasing students’ motivation towards PA and hence increase their PA levels in class (e.g., PE) and out of class (leisure time). The findings of this review should help researchers and practitioners, operating in health promotion and health education, to develop and implement interventions that effectively promote PA behavior based on increased motivation.

For this purpose, the findings of this review will be disseminated to academic and non-academic audiences through peer-reviewed publications, conferences, and formal presentations and in formal meetings.

## Additional files

Additional file 1: PRISMA-P (Preferred Reporting Items for Systematic review and Meta-Analysis Protocols) 2015 checklist: recommended items to include in a systematic review protocol. (DOC 84 kb)

Additional file 2: Search strategy. (DOCX 14 kb)

## Abbreviations

MVPA: Moderate-to-vigorous physical activity
PA: Physical activity
PE: Physical education
PRISMA: Preferred Reporting Items for Systematic Reviews and Meta-Analysis
PRISMA-P: Preferred reporting items for systematic review and meta-analysis protocols
SDT: Self-determination theory
