# Supplementary material for: Effects of school-based interventions on motivation towards physical activity in children and adolescents: protocol for a systematic review
Source: Syst Rev. 2019 May 10;8:113. doi: 10.1186/s13643-019-1029-1 (PMC6511217; doi:10.1186/s13643-019-1029-1)
Supplement: Supplementary file 2 — Search strategy. (DOCX 14 kb) [file 13643_2019_1029_MOESM2_ESM.docx]

Additional file 2. Search strategy

|  | Search terms |
| --- | --- |
|  | (child*) |
|  | (youth*) |
|  | (adolescent*) |
|  | (student*) |
|  | (pupil*) |
|  | (girl*) |
|  | (boy*) |
|  | (school*) |
|  | (physical education*) |
|  | (lesson*) |
|  | (physical activit*) |
|  | (exercise*) |
|  | (sport*) |
|  | (movement*) |
|  | (cycl*) |
|  | (walk*) |
|  | (intervention*) |
|  | (training*) |
|  | (experiment*) |
|  | (program*) |
|  | (education*) |
|  | (treatment*) |
|  | (evaluation*) |
|  | (motiv*) |
|  | (“self-determination*”) |
|  | (“intrinsic motiv*” |
|  | (“extrinsic motiv*”) |
|  | (“external regulation*”) |
|  | (“introjected regulation*”) |
|  | (“intrinsic regulation*”) |
|  | (introjection) |
|  | (“identified regulation*”) |
|  | (“integrated regulation*”) |
|  | (amotiv*) |
|  | (autonom*) |
|  | (competence*) |
|  | (relatedness*) |
|  | (enjoyment*) |
|  | (“basic* psycholog* need*”) |
|  | (satisfaction*) |
|  | (“motivation* climate*”) |
|  | 1 OR 2 OR 3 OR 4 OR 5 OR 6 OR 7 |
|  | 8 OR 9 OR 10 |
|  | 11 OR 12 OR 13 OR 14 OR 15 OR 16 |
|  | 17 OR 18 OR 19 OR 20 OR 21 OR 22 OR 23 |
|  | 24 OR 25 OR 26 OR 27 OR 28 OR 29 OR 30 OR 31 OR 32 OR 33 OR 34 OR 35 OR 36 OR 37 OR 38 OR 39 OR 40 OR 41 |
|  | 42 AND 43 AND 44 AND 45 AND 46 |
|  | Remove duplicates from 47 |
